# Unraveling 1,4-Butanediol Metabolism in *Pseudomonas putida* KT2440

**DOI:** 10.3389/fmicb.2020.00382

**Published:** 2020-03-17

**Authors:** Wing-Jin Li, Tanja Narancic, Shane T. Kenny, Paul-Joachim Niehoff, Kevin O’Connor, Lars M. Blank, Nick Wierckx

**Affiliations:** ^1^Institute of Applied Microbiology-iAMB, Aachen Biology and Biotechnology-ABBt, RWTH Aachen University, Aachen, Germany; ^2^UCD Earth Institute and School of Biomolecular and Biomedical Science, University College Dublin, Dublin, Ireland; ^3^BEACON – SFI Bioeconomy Research Centre, University College Dublin, Dublin, Ireland; ^4^Bioplastech Ltd., NovaUCD, Belfield Innovation Park, University College Dublin, Dublin, Ireland; ^5^Institute of Bio- and Geosciences IBG-1: Biotechnology, Forschungszentrum Jülich, Jülich, Germany

**Keywords:** laboratory evolution, *Pseudomonas putida*, proteomics, genomics, plastic upcycling

## Abstract

Plastics, in all forms, are a ubiquitous cornerstone of modern civilization. Although humanity undoubtedly benefits from the versatility and durability of plastics, they also cause a tremendous burden for the environment. Bio-upcycling is a promising approach to reduce this burden, especially for polymers that are currently not amenable to mechanical recycling. Wildtype *P. putida* KT2440 is able to grow on 1,4-butanediol as sole carbon source, but only very slowly. Adaptive laboratory evolution (ALE) led to the isolation of several strains with significantly enhanced growth rate and yield. Genome re-sequencing and proteomic analysis were applied to characterize the genomic and metabolic basis of efficient 1,4-butanediol metabolism. Initially, 1,4-butanediol is oxidized to 4-hydroxybutyrate, in which the highly expressed dehydrogenase enzymes encoded within the PP_2674-2680 *ped* gene cluster play an essential role. The resulting 4-hydroxybutyrate can be metabolized through three possible pathways: (i) oxidation to succinate, (ii) CoA activation and subsequent oxidation to succinyl-CoA, and (iii) beta oxidation to glycolyl-CoA and acetyl-CoA. The evolved strains were both mutated in a transcriptional regulator (PP_2046) of an operon encoding both beta-oxidation related genes and an alcohol dehydrogenase. When either the regulator or the alcohol dehydrogenase is deleted, no 1,4-butanediol uptake or growth could be detected. Using a reverse engineering approach, PP_2046 was replaced by a synthetic promotor (14g) to overexpress the downstream operon (PP_2047-2051), thereby enhancing growth on 1,4-butanediol. This work provides a deeper understanding of microbial 1,4-butanediol metabolism in *P. putida*, which is also expandable to other aliphatic alpha-omega diols. It enables the more efficient metabolism of these diols, thereby enabling biotechnological valorization of plastic monomers in a bio-upcycling approach.

## Introduction

Plastics, in all forms, are an ubiquitous cornerstone of modern civilization. They contribute greatly to a more efficient society, i.e., through the reduction of packaging weight, the increase in shelf life of foods, and the insulation of homes and refrigerators. Although humanity undoubtedly benefits from the versatility and durability of plastics, these characteristics also make them a tremendous burden for the environment. To reduce this impact, strategies beyond incineration, landfill and inefficient recycling are needed.

One of these approaches involves bio-upcycling, the microbial degradation of plastics and its conversion into value-added material (Wierckx et al., 2015; Narancic and O’Connor, 2017). Proofs of principle for the microbial conversion of selected plastics are already available. For instance, polyethyleneterephthalate (PET) was pyrolized and subsequently converted to polyhydroxyalkanoates (PHA) (Kenny et al., 2008; Kenny et al., 2012). A similar processes enabled conversion of polystyrene and polyethylene to PHA (Ward et al., 2006; Guzik et al., 2014). Polyurethanes (PU) are hardly amenable to mechanical recycling due to their molecular diversity and the fact that many PU are thermosets which can’t be molten and re-molded. PU are produced by reacting aliphatic or aromatic diisocyanates with polyols and α,ω-diols as chain extenders. Depending on the monomer composition and chain lengths, polymer properties are diverse, which is key for PU’s versatility. Applications can be found in paints and coatings, in building insulation and as sealants, as well as in flexible foams and absorbents for many end-user products like pillows and mattresses. In the context of a bio-upcycling strategy, bacteria and fungi have been found to degrade PU, including several Pseudomonads which grow on PU at high rates (Howard, 2002). A range of PU-degrading ester- and urethane hydrolases have been identified (Hung et al., 2016; Schmidt et al., 2017; Danso et al., 2019; Magnin et al., 2019a, b). Besides this, chemical recycling of PU is also possible with more mature technologies (Zia et al., 2007; Behrendt and Naber, 2009). In addition to the diamines, which are relatively valuable and can be extracted (Bednarz et al., 2017), typical PU monomers like adipic acid, 1,4-butanediol, and ethylene glycol are released during the process of depolymerization. Degradation pathways for ethylene glycol (Franden et al., 2018; Li et al., 2019) and adipic acid (Parke et al., 2001) are known. Yet, surprisingly little is known about the microbial catabolism of 1,4-butanediol.

1,4-butanediol is one of the major chain extenders used in the production of polyurethanes. It is also a common co-monomer in many polyesters such as polybutylene terephthalate and polybutylene adipate terephthalate. As commodity chemical, 1,4-butanediol is used to manufacture 2.5 million tons of plastics and polyesters (Yim et al., 2011). Additionally, it is used as a platform chemical to produce tetrahydrofuran and γ-butyrolactone, with a total market size valued at USD 6.19 billion in 2015 and is still growing (Grand View Research, 2017). So far, research was mainly focused on the sustainable production of 1,4-butanediol (Burgard et al., 2016). Its *de novo* microbial production was achieved in *E. coli* by identifying and implementing artificial routes for 1,4-butanediol biosynthesis (Yim et al., 2011). The verified and tested pathway starts with the TCA cycle intermediate succinyl-CoA. The heterologous CoA-dependent succinate semialdehyde dehydrogenase (SucD) from *Clostridium kluyveri* and either a native or heterologous 4-hydroxybutyrate dehydrogenase from *C. kluyveri*, *Porphyromonas gingivalis* or *Ralstonia eutropha* catalyze the reaction from succinyl-CoA to 4-hydroxybutyrate. After CoA activation, 4-hydroxybutyryl-CoA will be further reduced by alcohol and aldehyde dehydrogenases to the final product 1,4-butanediol. In addition to this commonly used pathway, alternative potential routes via α-ketoglutarate, glutamate or acetyl-CoA were described (Yim et al., 2011). Conversion of xylose to 1,4-butanediol has also been described (Liu and Lu, 2015).

Butanol is a substrate with structural similarities to 1,4-butanediol. Usually, butanol concentrations above 1–2% (135–270 mM) are toxic or at least growth-inhibiting for most of microbes, including *Pseudomonas putida* BIRD-1, DOT-T1E, and KT2440 (Cuenca et al., 2016). Nevertheless, *Pseudomonas* exhibits promising traits on tolerating, assimilating or at least surviving butanol (Rühl et al., 2009). To cope with butanol, classic solvent defense mechanisms like efflux pumps, membrane modifications or rebalancing of the redox state are activated (Ramos et al., 2002; Basler et al., 2018). Further, *P. putida* KT2440 is capable of rapid butanol oxidation to butyrate via a variety of alcohol- and aldehyde dehydrogenases (Simon et al., 2015; Vallon et al., 2015; Cuenca et al., 2016). Prominent among these are PedE, PedH, and PedI alcohol and aldehyde dehydrogenases, encoded in the so-called ped cluster. These have a highly relaxed substrate specificity and are capable of oxidizing, among others, ethanol, phenylethanol, butanol, and butanal (butyraldehyde) (Wehrmann et al., 2017). The resulting butyrate is CoA-activated by acyl-CoA synthetases like AcsA1 (PP_4487), and subsequently undergoes β-oxidation.

Non-pathogenic Pseudomonads have an established track record in bioremediation and biodegradation processes (Samanta et al., 2002; Spini et al., 2018; Tahseen et al., 2019), and different strains of this genus are also suitable candidates to perform bio-upcycling (Kenny et al., 2008; Wierckx et al., 2015; Wilkes and Aristilde, 2017). One of the widely used biotechnological hosts is *P. putida* KT2440, which possesses extensive metabolic abilities (Nelson et al., 2002; Nikel et al., 2014; Nikel and de Lorenzo, 2018). Being a soil bacterium and therefore exposed to different environmental surroundings, it is equipped with tolerances and metabolic capabilities toward a broad spectrum of substances. The 6.18 Mb genome of *P. putida* KT2440 harbors a broad spectrum of oxygenases, oxidoreductases as well as hydrolases, transferases, and dehydrogenases (Belda et al., 2016). This wide range of enzymes enables *P. putida* KT2440 to modify an abundance of alcohols and aldehydes (Wierckx et al., 2011).

In this work, *P. putida* KT2440 strains with an enhanced growth rate on 1,4-butanediol are obtained by adaptive laboratory evolution (ALE), and analyzed by proteomics and genome resequencing in order to determine possible degradation routes. The improved growth phenotype was subsequently reverse-engineered into the wildtype, thereby generating a deeper understanding of 1,4-butanediol metabolism and broadening the applicability of *P. putida* for plastic upcycling.

## Materials and Methods

### Chemicals, Media, and Cultivation Conditions

The chemicals used in this work were obtained from Carl Roth (Karlsruhe, Germany), Sigma-Aldrich (St. Louis, MO, United States), or Merck (Darmstadt, Germany) unless stated otherwise. Glycerol was kindly provided by Bioeton (Kyritz, Germany).

All strains used in this work are listed in Table 1. Cultivations were performed in LB – complex medium (10 g L^–1^ tryptone, 5 g L^–1^ yeast extract and 5 g L^–1^ sodium chloride) or, for quantitative microbiology experiments, in mineral salt medium (MSM) (Hartmans et al., 1989), solidified when needed with 1.5% agar (w/v), containing different amount of C source. Precultures were supplied with 20 mM glucose, whereas 20 mM 1,4-butanediol were used for studies with 1,4-butanediol.

**TABLE 1:** Pseudomonas putida strains used in this work with listed genotype and references.

No.	Strain	Genotype	References
1	KT2440 wildtype	Cured, restriction-deficient derivative of *P. putida* mt-2	Bagdasarian et al., 1981
2	B10.1	KT2440 ALE in BDO, single strain A6	This work
3	B10.2	KT2440 ALE in BDO, single strain C2	This work
4	KT2440 ΔPP_2046	ΔPP_2046 in KT2440	This work
	B10.1 ΔPP_2046	ΔPP_2046 in B10.1	This work
	B10.2 ΔPP_2046	ΔPP_2046 in B10.2	This work
5	KT2440 ΔPP_2046:14g	ΔPP_2046:14g in KT2440	This work
	B10.1 ΔPP_2046:14g	ΔPP_2046:14g in B10.1	This work
	B10.2 ΔPP_2046:14g	ΔPP_2046:14g in B10.2	This work
6	KT2440 Δped	ΔpedE-I in KT2440	Li et al., 2019
7	KT2440 ΔpedE	ΔpedE in KT2440	This work
	KT2440 ΔpedH	ΔpedH in KT2440	This work
8	KT2440 ΔpedI	ΔpedI in KT2440	This work
9	KT2440 ΔPP_2047-2051	knockout PP_2047-51 in KT2440	This work
	B10.A ΔPP_2047-2051	knockout PP_2047-51 in B10.A	This work
	B10.B ΔPP_2047-2051	knockout PP_2047-51 in B10.B	This work
10	KT2440 ΔPP_2049	ΔPP_2049 in KT2440	This work
	B10.1 ΔPP_2049	ΔPP_2049 in B10.1	This work
	B10.2 ΔPP_2049	ΔPP_2049 in B10.2	This work
11	KT2440 ΔPP_2051	ΔPP_2051 in KT2440	This work
	B10.1 ΔPP_2051	ΔPP_2051 in B10.1	This work
	B102 ΔPP_2051	ΔPP_2051 in B10.2	This work
	KT2440 ΔPP_0411-13	ΔPP_0411-13	This work
	B10.1 ΔPP_0411-13	ΔPP_0411-13	This work
	B10.2 ΔPP_0411-13	ΔPP_0411-13	This work

For plasmid maintenance, *E. coli* strains and *P. putida* KT2440 strains were cultivated in media supplemented with 50 mg L^–1^ kanamycin, which was sterilized by using a 0.2 μm syringe filter (Carl Roth GmbH + Co. KG, Karlsruhe, Germany).

Liquid cultivations were incubated at 30°C for *Pseudomonas* and 37°C for *E. coli*, 200 rpm shaking speed with an amplitude of 50 mm in a Multitron shaker (INFORS, Bottmingen, Switzerland) using 100 mL non-baffled Erlenmeyer flasks with metal caps, containing 10 mL culture volume for a pre-culture and 500 mL non-baffled Erlenmeyer flasks with metal caps, containing 50 mL culture volume for a main culture.

For online growth detection, 96-well plates with 200 μL or 24-well plates with 4–3 mL culture volume were inoculated with a pre-culture containing 4–3 mL MSM with 20 mM glucose in 24-well System Duetz plates (Enzyscreen, Heemstede, The Netherlands), cultivated in a Multitron shaker (INFORS, Bottmingen, Switzerland) with a 300 rpm shaking speed with an amplitude of 50 mm. Inoculated Growth Profiler^®^ plates were incubated at 30°C, 225 rpm shaking speed with an amplitude of 50 mm in the Growth Profiler^®^ 960 (Enzyscreen, Heemstede, The Netherlands).

Adaptive laboratory evolution (ALE) was performed as follows: a pre-culture of *P. putida* KT2440, cultivated in MSM with 20 mM glucose, was used to inoculate 250 mL clear glass Boston bottles with Mininert valves (Thermo Fisher Scientific, Waltham, MA, United States) containing 20 mM 1,4-butanediol for the adaptation on 1,4-butanediol (final OD_600_ of 0.01). Serial transfers were reinoculated several times after the cultures reached an OD_600_ of at least 0.5, with a starting OD_600_ of 0.1. After growth was detected (usually overnight), single colonies were isolated from ALE cultures by streaking samples on LB agar plates. After ALE on 1,4-butanediol, two strains (B10.1 and B10.2) out of 72 strains were selected according to their growth behavior in MSM with 20 mM 1,4-butanediol determined using the Growth Profiler^®^ 960 (Enzyscreen, Heemstede, The Netherlands).

Growth experiments for PHA accumulation were carried out in 250 mL Erlenmeyer flasks containing 50 mL of nitrogen limited MSM medium supplemented with 80 mM 1,4 butanediol. Nitrogen limited MSM medium contains 9 g/L Na_2_HPO_4_.12H_2_0, 1.5 g/L KH_2_PO_4_, 0.25 g/l NH_4_Cl, trace elements. An overnight culture was prepared by inoculating 2 mL of medium with a single colony from a plate and incubating overnight at 30°C and shaking at 200 rpm. Five hundred micro liter of the 2 mL MSM overnight culture was used as an inoculum for the 50 mL cultures which were incubated under the same conditions for 48 h. Octanoic acid (20 mM) was added to some flasks after 24 h.

### Molecular Work

#### DNA Procedures

The construction of plasmids was performed either by standard restriction-ligation or Gibson assembly (Gibson et al., 2009) using the NEBuilder HiFi DNA Assembly (New England Biolabs, Ipswich, MA, United States). DNA modifying enzymes were purchased from New England Biolabs, for dephosphorylation Fast AP Thermo Sensitive Alkaline Phosphatase (Thermo Fisher Scientific, Langenselbold, Germany) was used. Primers were purchased as unmodified DNA oligonucleotides from Eurofins Genomics (Ebersberg, Germany) and are listed in Supplementary Table S1. Clonal DNA sequences were amplified using the Q5 High-Fidelity Polymerase (New England Biolabs, Ipswich, MA, United States). DNA- ligations were performed by using T4 ligase from Fermentas (Thermo Fisher Scientific, Langenselbold, Germany) according to the protocol. Arbitrary-primed PCR was performed as described by Martínez-García et al. (2014). For the transformation of DNA assemblies and purified plasmids (Supplementary Table S2) into competent *E. coli* a heat shock protocol was performed (Hanahan, 1983). For *P. putida* transformations either conjugational transfer or electroporation were performed as described by Wynands et al. (2018). Knockout strains were obtained using the pEMG system described by Martínez-García and de Lorenzo (2011) with a modified protocol described by Wynands et al. (2018). Plasmid inserts and gene deletions were confirmed by Sanger sequencing performed by Eurofins Genomics (Ebersberg, Germany).

In order to perform PCR directly from bacteria the alkaline polyethylene glycerol-based method was used (Chomczynski and Rymaszewski, 2006). Therefore, cell material was picked and dissolved in 50 μL of the reagent, containing 60 g PEG 200 with 0.93 mL 2 M KOH and 39 mL water, with a pH of 13.4. After incubation for 3–15 min, 2 μL of the sample was used as template in a 25 μL PCR reaction.

### Analytical Methods

#### Growth Monitoring Methods

Bacterial growth was monitored as optical density at a wavelength of λ = 600 nm (OD_600_) with an Ultrospec 10 Cell Density Meter (GE Healthcare, Little Chalfont, Buckinghamshire, United Kingdom). Growth rates (μ) are determined by fitting an exponential curve to a plot of OD_600_ over time of a culture in the exponential phase. The online analysis of growth using the Growth Profiler^®^ was analyzed using the Growth Profiler^®^ Control software V2_0_0. Cell densities are expressed as G-value, which is derived from imaging analysis of microtiter plates with transparent bottoms.

#### PHA Analysis

Cells were harvested by centrifugation at 3320 × *g* for 10 min and then lyophilized and weighed for determination of cell dry weight. PHA content was determined by subjecting lyophilized cells to acidic methanolysis (Brandl et al., 1988; Lageveen et al., 1988). Five to ten milligram of dried cells were resuspended in 2 mL of acidified methanol (15% H_2_SO_4_, v/v) and 2 mL of chloroform containing 6 mg/l benzoate methyl ester as an internal standard. The solution was placed in 15 mL Pyrex test tubes, sealed and incubated at 100°C for 3 h. The tubes were then placed on ice for 1 min. One milliliter of water was added to each tube and the solution mixed by vigorous vortexing. The phases were allowed to separate, and the organic phase was removed and passed through a filter before further analysis.

The 3-hydroxyalkanoic acid methyl esters were analyzed by gas chromatography (GC) using an Agilent 6890N chromatograph equipped with a HP Innowax column (30 m × 0.25 mm × 0.5 μm) and a flame ionization detector (FID). An oven ramp cycle was employed as follows, 120°C for 5 min, increasing by 3°C/min to 180°C, 180°C for 10 min. A 20:1 split was used with helium as the carrier gas and an inlet temperature of 250°C. Commercially available 3-hydroxyalkanoic acids (Bioplastech Ltd., Dublin, Ireland) were methylated as described above for PHA samples and used as standards to identify PHA monomers.

#### Extracellular Metabolites

For measuring extracellular metabolites, samples taken from liquid cultivation were centrifuged for 3 min at 17,000 × *g* to obtain supernatant for High-Performance Liquid Chromatography (HLPC) analysis using a Beckman System Gold 126 Solvent Module equipped with a Smartline 2300 refractive index detector (Knauer, Berlin, Germany). Analytes were eluted using a 300 × 8 mm organic acid resin column together with a 40 × 8 mm organic acid resin precolumn (both from CS Chromatographie, Langerwehe, Germany) with 5 mM H_2_SO_4_ as mobile phase at a flow rate of 0.7 mL min^–1^ at 70°C (Li et al., 2019).

#### Genome Sequencing

Genomic DNA for resequencing was isolated through a High Pure PCR Template Preparation Kit (ROCHE life science, Basel, Switzerland). Sequencing and SNP/InDel (single nucleotide polymorphism/insertion and deletion polymorphism) calling was done by GATC (Konstanz, Germany) using Illumina technology as paired-end reads of 125 base pairs. To map the reference sequence against the database, BWA with default parameters was used (Li and Durbin, 2009). SNPs and InDels, analyzed by GATK’s UnifiedGenotyper (McKenna et al., 2010; DePristo et al., 2011), were listed and visualized with the Integrative Genomics Viewer (IGV) (Thorvaldsdóttir et al., 2013).

The sequences have been deposited in the NCBI Sequence Read Archive (SRA) with the accession number SRP148839 for ethylene glycol ALE strains (including our laboratory wildtype SRX4119395 used in this study) and SRP148839 for the 1,4-butanediol ALE strains.

#### Proteomics

The evolved strains B10.1 and B10.2 were cultivated along with the wild type *P. putida* KT2440 in 50 mL MSM medium supplemented with 20 mM 1,4-butanediol or 13 mM glucose (both equivalent to 80 mM C). The cultures were harvested by centrifugation and prepared for proteomic analysis as previously described (Narancic et al., 2016). Samples were sent to T. Narancic at University of Dublin to perform the following protocol. For total protein concentrations, peptide fragments obtained by trypsin digestion were analyzed on the Q-Exactive Hybrid Quadrupole Orbitrap Mass Spectrometer (MS; Thermo Fisher Scientific) connected to a Dionex Ultimate 3000 (RSLCnano; Thermo Fisher Scientific) chromatography system (Buffer A: 97% water, 2.5% acetonitrile, 0.5% acetic acid; buffer B: 97% acetonitrile, 2.5% water, 0.5% acetic acid; all solvents were LC-MS grade). The mass spectrometer was operated in positive ion mode with a capillary temperature of 320°C and a potential of 2300 V applied to the frit. All data were acquired with the MS operating in automatic data-dependent switching mode. A high-resolution (70,000) MS scan (300–1600 m/z) was performed using the Q Exactive to select the 12 most intense ions prior to MS/MS analysis using HCD. The identification and quantification were performed using the Andromeda peptide identification algorithm integrated into MaxQuant (Cox and Mann, 2008; Cox et al., 2011). *P. putida* KT2440 protein sequence database downloaded from UniProt^1^ in April 2016 was used as a reference (The UniProt Consortium, 2019). Label-free quantification (LFQ) was used to compare the expression level of proteins across samples and growth conditions (Wang et al., 2003). Proteins with a twofold change or higher and a significant change in *t*-test (FDR 0.01) were automatically accepted, while spectra with no specific change were manually checked for quality.

Each sample had three biological replicates, and each biological replicate was then prepared for the proteomic analysis as a technical replicate. Statistical analysis was performed using Perseus and built-in Welche’s *t*-test with FDR set at 0.01 (Tyanova et al., 2016). The proteins with at least twofold change were functionally annotated using David bioinformatics (Huang et al., 2009a, b) and clustered into orthologous groups using EggNOG (Huerta-Cepas et al., 2016).

#### Detection of Dehydrogenase Activity

To perform the enzyme assay, cells from a pre-culture were used to inoculate the main culture containing 20 mM glucose and 5 mM 1,4-butanediol. After 16 h of cultivation, crude extract was isolated using BugBuster (Merck, Darmstadt, Germany) and was desalted using PD-desalting columns (GE Healthcare, Buckinghamshire, United Kingdom) and eluted in 100 mM glycinglycin buffer. Protein concentrations were estimated by standard Bradford test at 595 nm. For the dehydrogenase assay, a modified protocol from Kagi and Vallee (1960) was followed, in which 5 mM 4-hydroxybutyrate or 4% ethanol as control were used as substrate. The formation of NADH was measured at 340 nm in a 96-well-plate at 30°C in a well plate reader from Synergy Mx from Biotek (Bad Friedrichshall, Germany). To obtain a homogeneous mixture, after the addition of NAD^+^ or 4-hydroxybutyrate, the well-plate was shaken for 3 s at highest speed available.

#### Statistics

Statistical probability values were, if not stated otherwise, calculated using a paired Student’s t-distribution test with homogeneity of variance (*n* = 3, significance level of 0.05). In case of duplicates, errors are expressed as deviation from the mean (*n* = 2).

## Results

### Isolation of Strain With Enhanced Growth on 1,4-Butanediol by ALE

**FIGURE 1:**
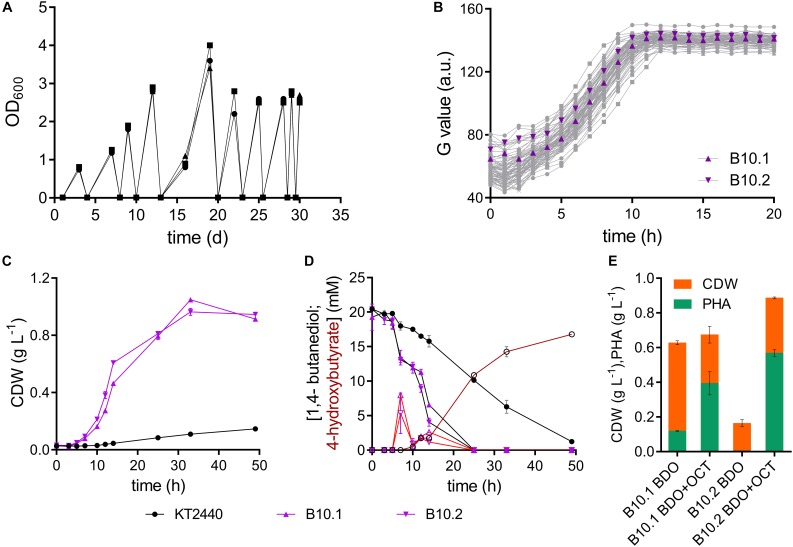
Adaptive laboratory evolution of *P. putida* KT2440 on 1,4-butanediol. **(A)** Three parallel sequential batch cultivations on MSM with 20 mM 1,4-butanediol. **(B)** Growth of single strains isolated from each ALE batch on MSM with 20 mM 1,4-butanediol. The strains B10.1 (purple triangle) and B10.2 (purple inverted triangle) were selected for further investigation. Growth was detected via a Growth Profiler^®^ using a 96-well plate. **(C)** Biomass growth and **(D)** 1,4-butanediol (closed symbols, black lines) and 4-hydroxybutyrate (open symbols, red lines) measured in cultures of the wildtype and evolved strains B10.1 and B10.2 in MSM with 20 mM 1,4-butanediol. **(E)** Growth and PHA formation of strains B10.1 and B10.2 cultivated for 48 h in nitrogen-limited MSM medium with 80 mM 1,4-butanediol (BDO), supplemented with or without 20 mM octanoic acid (OCT) after 24 h. Cultures took place in 250 mL Erlenmeyer shake flasks with a culture volume of 50 mL. Error bars indicate the deviation of the mean (*n* = 2).

To study the growth of *P. putida* KT2440 and possible intermediate production when metabolizing 1,4-butanediol, growth experiments in shake flasks were performed. The wildtype showed poor growth (μ_max_ = 0.082 ± 0.004 h^–1^) on MSM with 20 mM 1,4-butanediol, requiring more than 50 h to consume all substrate (*t* = 49; 1.3 ± 0.1 mM), while secreting high levels of the oxidation product 4-hydroxybutyrate (*t* = 49 h; 16.8 ± 0.3 mM) (Figure 1). This slow growth implies that in principle the metabolic routes are present in *P. putida* KT2440, but they are not operating optimally under the chosen conditions.

To enhance its ability to grow on 1,4-butanediol, wildtype *P. putida* KT2440 was subjected to ALE. This method is known to enable the selection of mutated strains with enhanced properties toward specific environments, likely affecting transcriptional regulatory systems (Dragosits and Mattanovich, 2013; Lennen et al., 2019; Li et al., 2019). Cultures of *P. putida* KT2440 were serially re-inoculated to fresh media containing 20 mM 1,4-butanediol ten times, as soon as growth was observed in form of optical densities above 0.8 (Figure 1). All three parallel evolution lines grew with the same trend. While the first three batches reached an OD_600_ between 0.8 and 2 after 3–4 days, later batches reached an OD_600_ between 2.5 and 3.5 after 2 days or less. The ALE was stopped after approximately 47 generations, when growth on 1,4-butanediol reached an OD_600_ of 2.5 overnight. Single colonies were isolated by streaking the three ALE cultures on LB plates. From each of the three evolution lines, 24 single colonies were tested for growth on 1,4-butanediol in a 96- well plate using a Growth Profiler^®^ (Figure 1). Overall, the growth of these single clones was relatively similar, indicating a rather homogeneous evolved population. The two strains with the highest growth rates were selected, from different evolutionary lines, according to their growth in MSM with 20 mM 1,4-butanediol. These isolated clonal strains are named B10.1 and B10.2 (Figure 1).

The two evolved strains grew faster on 1,4-butanediol than the wildtype, even after several generations in complex medium or MSM containing glucose, indicating that the observed phenotype was evolutionary fixed in the genome. The single evolved isolates B10.1 and B10.2 reach growth rates of 0.33 ± 0.054 h^–1^ and 0.31 ± 0.001 h^–1^, respectively. In contrast to the wildtype they completely consume all carbon source, reaching maximum biomass concentrations of 1.05 ± 0.0 g_*cdw*_ L^–1^ and 0.96 ± 0.02 g_*cdw*_ L^–1^ after 33 h. This translates to an average biomass yield of 0.56 ± 0.025 g/g for the evolved strains. This relatively high yield is likely caused by the high degree of reduction of butanediol, providing considerable reducing equivalents especially in the initial oxidation reactions (Li et al., 2019). In contrast to the wildtype, the evolved strains only transiently accumulate low concentrations of 4-hydroxybutyrate, which are rapidly metabolized within 4 h (Figure 1). All 1,4-butanediol and derivatives that could be detected by HPLC were consumed within 25 h, although biomass still increased significantly beyond this point. Other intermediates not detected by HPLC, possibly the lactone of 4-hydroxybutyrate, could likely accumulate transiently in the cultures of the evolved strains. However, the high biomass yield suggests that all carbon source was consumed at the end of the culture.

In order to assess the applicability of these evolved strains in a bio-upcycling approach, they were cultured in a nitrogen-limited MSM medium with 80 mM 1,4-butanediol. Further cultures were supplemented with 20 mM octanoic acid after 24 h. These conditions enable the production of polyhydroxyalkanoate (PHA) from a (co-)feed of 1,4-butanediol. Without octanoic acid co-feed, strain B10.1 strains reached a final biomass concentration of 0.63 ± 0.11 g L^–1^, of which 19% (0.12 ± 0.003 g L^–1^) is PHA. Surprisingly, strain B10.2 reached a much lower biomass density, with only 3% PHA. With an octanoic acid co-feed, strain B10.2 reached the highest biomass concentrations of 0.89 ± 0.005 g L^–1^, of which 64% (0.57 ± 0.02 g L^–1^) is PHA (Figure 1). This proves that 1,4-butanediol can be used as a (co-)substrate for the production of a value-added biopolymer, thereby in principle enabling the upcycling of e.g., hydrolyzed PU or polyester waste.

Evolution successfully yielded strains with a 4- or 3.7-fold improved growth rate on 1,4-butanediol compared to the wildtype. The fact that the wildtype accumulates much more 4-hydroxybutyrate than the evolved strains indicates that this is likely the main metabolic bottleneck which was affected by ALE.

### Systems Analysis of 1,4-Butanediol Degradation in *P. putida* KT2440

To investigate the molecular basis of their enhanced growth on 1,4-butanediol, the genomes of the evolved strains B10.1 and B10.2 were resequenced (NCBI SRA accession number SRP148839). The sequences were compared to our laboratory wildtype (SRX4119395) and a reference database genome of *P. putida* KT2440 (Belda et al., 2016, AE015451.2). A comparison of the latter two was previously described in the context of ethylene glycol metabolism in *P. putida* (Li et al., 2019). Therefore, we focus here only on differences between our laboratory wildtype and the B10 strains. In the evolved strains B10.1 and B10.2, seven and eight mutations, respectively, were identified in addition to the mutations already present in the laboratory wildtype. Most of these mutations were either silent or intergenic. In addition to these, in the genome of B10.1, an in-frame deletion of 69 bp was found in PP_2139, encoding DNA topoisomerase I. Since this enzyme is related to DNA replication and repair (Wang, 2002), this alteration is unlikely to affect 1,4-butanediol metabolism specifically. However, this mutation might still be favorable in a general sense by affecting growth rate through DNA replication. Furthermore, a missense mutation was identified B10.2 that affects PP_2889, encoding the transmembrane anti-sigma factor PrtR (Calero et al., 2018; Supplementary Table S3). An amino exchange (A240G, GCG/GGG) in this regulator, involved in temperature-related protease production (Burger et al., 2000), might enhance tolerance toward 1,4-butanediol and its oxidation products. Both of these mutations are likely related to general ALE effects selecting for faster growth or higher tolerance to chemical stressors, rather than affecting the operation of the metabolic network.

The PP_2046 gene, encoding for a LysR-type transcriptional regulator, stood out for being mutated in both evolved strains, with each carrying a different mutation. In B10.1, a nonsense mutation caused the loss of the start codon (ATG/ATA), while in B10.2 a missense mutation caused an amino acid exchange (E34G, GAG/GGG) in the helix-turn-helix DNA binding domain of the regulator (Supplementary Figure S1 and Supplementary Table S3). The mutations in this regulator likely affect the expression of the adjacent operon PP_2047-51 which encodes an iron-containing alcohol dehydrogenase, as well as enzymes involved in β-oxidation (Li et al., 2019).

In addition to the analysis of the changes on the genome level, proteomic analysis of the evolved strains and the wildtype during growth on glucose and 1,4-butanediol was conducted. This was done to identify relevant enzymes that are either constitutively expressed or natively induced by 1,4-butanediol. Three biological samples from each strain and culture condition, either grown on glucose or 1,4-butanediol in MSM, were harvested at mid-log phase (Supplementary Figure S2). The samples were normalized using total protein concentration to give the same starting protein concentration for all replicates.

In total, 2122 proteins were identified across all samples and growth conditions, representing 40% of the *P. putida* KT2440 proteome. The identified proteins exhibited a wide range of annotated biophysical (molecular mass, isoelectric point), biochemical (functional annotations) and structural (domains) properties, suggesting that the analysis was not biased in favor of, or against, any protein class.

**FIGURE 2:**
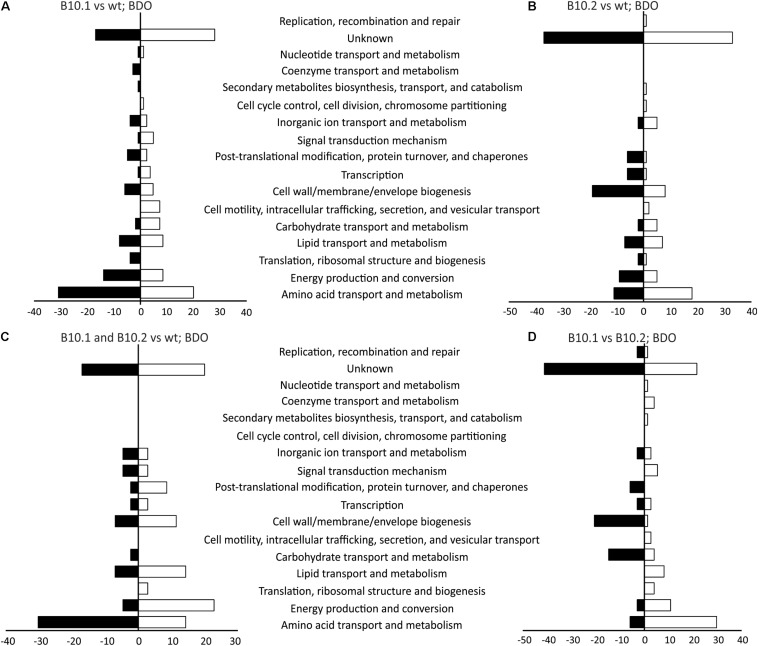
Proteins with significantly different levels of expression between the evolved strain B10.1 and wildtype *P. putida* KT2440 **(A)**, the evolved strain B10.2 and wildtype *P. putida* KT2440 **(B)**, the proteins showing the same trend of upregulation or downregulation in both evolved strains compared to the wild type **(C)**, and the proteins with different levels of expression in B10.1 and B10.2 **(D)** when grown with 1,4-butanediol (BDO) as a carbon and energy substrate. The number of proteins showing ≥ 2-fold change (*T*-test, FDR 0.01) in expression are given as clusters of orthologous groups (COG).

When cultivated on 1,4-butanediol the evolved strain B10.1 expressed 19 proteins which were not present in the wildtype, while 313 proteins were up- or downregulated at least two-fold compared to the wildtype. When evolved strain B10.2 was compared to the wildtype, 138 proteins showed at least two-fold difference in expression. The two evolved strains differed in their expression of 126 proteins. A large fraction of the differentially expressed proteins have no known function (Figure 2 and Supplementary Data Files 2). The second-largest group can be categorized in amino acid metabolism and transport according to the clusters of orthologous groups (COG) classification (Tatusov et al., 1997). The likely reason for this large number of proteins with different expression levels is a large difference in growth rate of the B10 strains and the wildtype on 1,4-butanediol and the turnover of proteins during growth.

**FIGURE 3:**
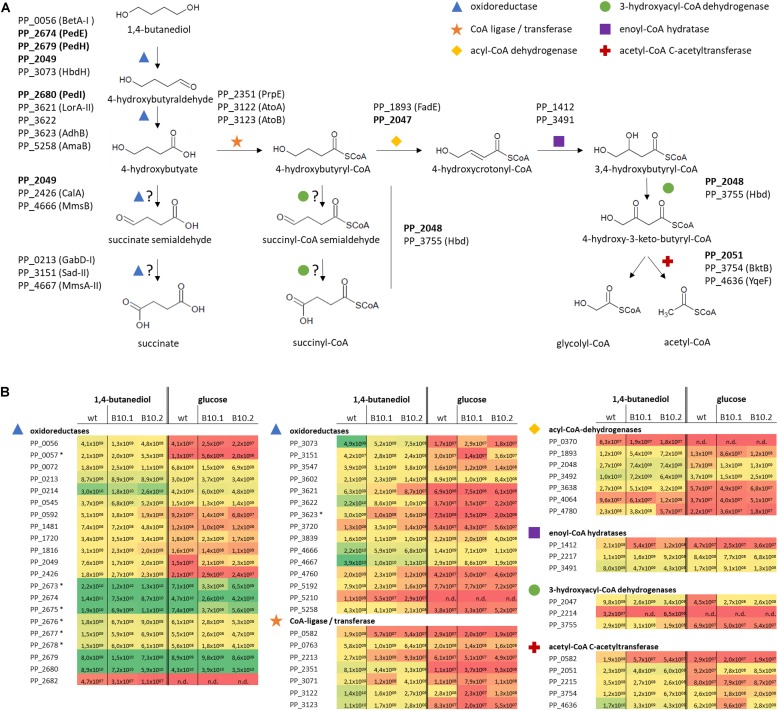
Hypothetical pathways for 1,4-butanediol metabolism **(A)** and expression levels (Label Free Quantification, LFQ; Tyanova et al., 2016) representing the sum of the ion signal recorded in the mass spectrometer for all peptides derived from each protein of the corresponding proteins **(B)**. Colors correspond to LFQ levels, red for low, yellow for average, and green for high values. Proteins which are strongly upregulated in response to growth on MSM with 1,4-butanediol compared to growth with glucose, or which have high expression level in all tested conditions, are indicated in **(A)**, with proteins further investigated in this work in bold. Protein expression levels **(B)** of selected proteins for *P. putida* KT2440 and the evolved strains B10.1 and B10.2 growing in MSM with glucose or 1,4-butanediol are listed as oxidoreductases (blue triangle), CoA-ligases (orange star), acyl-CoA dehydrogenases (yellow diamond), 3-hydroxyacyl-CoA dehydrogenase (green circle), enoyl-CoA hydratases (purple rectangle), and acetyl-CoA C-actyltransferase (red cross). n/d, not detected. In case of operons, associated transporters or accessory proteins are included, marked with *.

The top three highest expressed proteins during growth on glucose as well as 1,4-butanediol were PedE (ethanol dehydrogenase – PP_2674), PedI (aldehyde dehydrogenase – PP_2680) and Tu-B (PP_0452), an elongation factor which is involved in the regulation of protein synthesis by mediating aminoacyl tRNA into a free site of ribosomes (Noel and Whitford, 2016). The latter Tu-B is a general growth-associated protein (Klumpp et al., 2009). The former two proteins are encoded within the ped cluster (PP_2663-80) (Li et al., 2019). To focus on 1,4-butanediol metabolism, specific proteins with activities in putative catabolic pathways (Figure 3) and associated transport steps were further investigated.

Genome sequencing uncovered mutations in PP_2046. The corresponding protein was not detected in the proteome analysis, indicating no or a low basal expression below the detection limit of the applied method, which is not uncommon for transcriptional regulators. Proteins encoded by the downstream β-oxidation-related operon were strongly upregulated in the wildtype grown on 1,4-butanediol vs. glucose, including a 3-hydroxyacyl-CoA dehydrogenase (PP_2047, 22-fold), an acyl-CoA dehydrogenase (PP_2048, 16-fold), an iron-containing alcohol dehydrogenase (PP_2049, 52-fold), and an acetyl-CoA acetyltransferase (PP_2051, 25-fold). The hypothetical protein PP_2050 was not detected. On top of this strong induction by 1,4-butanediol in the wildtype, the genes in this operon were even further induced by 2.4- to 3-fold in the evolved strains compared to the wildtype (Figure 3 and Supplementary Data Files 2).

Theoretically, 1,4-butanediol can be metabolized through three possible pathways, all branching off at the point of 4-hydroxybutyrate. This 4-hydroxybutyrate was rapidly formed in cultivations of wildtype *P. putida* KT2440 on 1,4-butanediol, and also accumulates transiently with the B10 strains (Figure 1). This shows that oxidation of 1,4-butanediol to 4-hydroxybutyrate via alcohol and aldehyde oxidases already occurs at a high rate in the wildtype. The high expression levels of PedE, PedH and PedI suggest that these enzymes are major players in these oxidation steps. Although the encoding genes were not affected by the ALE, and they are only marginally upregulated when the strains were grown with 1,4-butanediol in comparison with growth on glucose, they are constitutively expressed on a very high level. This indicates a considerable metabolic investment of *P. putida* to be prepared for alcohol and aldehyde oxidation. This rapid oxidation is especially important for tolerance against the highly toxic aldehydes (Franden et al., 2018). Apparently, *P. putida* encounters such aldehydes often enough in its native environment to warrant this high constitutive expression. Besides these two enzymes, a number of other oxidoreductases are strongly induced upon growth on 1,4-butanediol vs. glucose. These include the GMC family oxidoreductase PP_0056 (BetA-I, along with its associated transporter PP_0057), the iron-containing alcohol dehydrogenase PP_2049, the 3-hydroxybutyrate dehydrogenase PP_3073 (HbdH), the isoquinoline oxidoreductase PP_3621-3 (IorAB-adhB), and the aldehyde dehydrogenase PP_5258 (amaB).

The resulting 4-hydroxybutyrate can theoretically be further oxidized by the same enzymes. However, the presence of a negatively charged carboxylate moiety likely affects substrate binding and it is more plausible that one or several other oxidoreductases with annotated activities on molecules with a carboxylate group similar to 4-hydroxybutyrate perform these oxidations. Several of such enzymes were upregulated during growth on 1,4-butanediol vs. glucose, including a 3-hydroxyisobutyrate dehydrogenase (PP_4666, MmsB) and a methylmalonate semialdehyde dehydrogenase (PP_4667 MmsA-II) (Steele et al., 1992; Zhou et al., 2013) for the alcohol oxidation step. The resulting succinate semialdehyde can be oxidized by the annotated succinate semialdehyde dehydrogenases PP_0213 (GabD-I) and PP_3151 (SadI), but also possibly by the methylmalonate-semialdehyde dehydrogenase PP_4667 (MmsA-II). The resulting oxidation product succinate can be further metabolized in the TCA cycle. The low growth rate of the wildtype indicates that, although multiple putative proteins for both activities are expressed at a high level, these latter two oxidations only occur at a low rate at best, with a likely bottleneck in the oxidation of 4-hydroxybutyrate.

**FIGURE 4:**
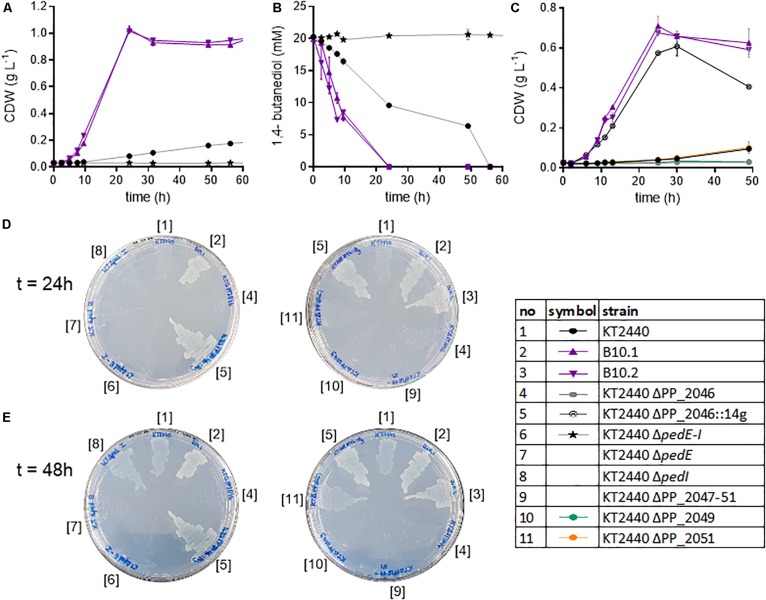
Analysis of knockout strains of *P. putida* KT2440. Biomass growth **(A)** and 1,4-butanediol concentrations **(B)** of the wildtype, evolved strains and the Δ*pedE-I* knockout cultivated in shake flasks in MSM with 20 mM 1,4-butanediol. Growth of selected strains on 20 mM 4-hydroxybutyrate **(C)**. Growth of selected strains on MSM agar plates with 20 mM 1,4-butanediol after 24 h **(D)** and 48 h **(E)**. Strain numbers next to the plates correspond to full strain names listed in Table 1. The contrast of the images was increased by 20% to improve visibility. Error bars indicate the deviation of the mean (*n* = 2).

As an alternative hypothesis to this direct oxidation route, 4-hydroxybutyrate can also be CoA-activated via CoA ligases or transferases. After CoA activation, 4-hydroxybutyryl-CoA can undergo β-oxidation, possibly through enzymes encoded by the PP_2047-51 operon downstream of PP_2046, which would result in glycolyl-CoA and acetyl-CoA (Figure 4). Other β-oxidation related proteins were also identified by the proteome analysis. Glycolyl-CoA may be converted into glycolate and subsequently metabolized through native pathways of *P. putida* (Li et al., 2019). Theoretically, 4-hydroxybutyryl-CoA could also be further oxidized by acyl-CoA dehydrogenases to generate succinyl-CoA, provided that these have a side activity on the 4-hydroxy group instead of their usual 3-hydroxylated substrates.

Also of note was the relatively strong differential expression of genes related to the metabolism and transport of amines. The PP_0411-4 operon was highly expressed in the wildtype, but not in the evolved strains, during growth on 1,4-butanediol (Supplementary Data Files 2). This operon encodes a polyamine ABC transporter for spermidine and putrescine, which are structurally and chemically similar to 1,4-butanediol. In spite of this large differential expression, no genomic mutations were found in the evolved strains surrounding the operon, and the knockout of PP_0411-14 in *P. putida* KT2440 did not influence growth on 1,4-butanediol (data not shown). In contrast, operons encoding metabolic pathways for 4-aminobutanoate (PP_2013-15), ethanolamine (PP_0542-44), and ornithine (PP_0999-1001) were strongly upregulated on 1,4-butanediol vs. glucose (Supplementary Data Files 2). The metabolism of some of these amines shares metabolic intermediates with the putative 1,4-butanediol pathways (Bandounas et al., 2011). Possibly, the high accumulation of 4-hydroxybutyrate in the wildtype induced the expression of these genes, leading to a misregulation during growth on 1,4-butanediol. Alternatively, one of the aldehyde intermediates may undergo amination, or the diamine transporter may facilitate uptake of 1,4-butanediol or its oxidation products.

### Pathway Validation

The abovementioned genomic and proteomic analyses indicate several possible genes and enzymes that are either natively expressed at a high level, upregulated in the presence of 1,4-butanediol, or activated by ALE. To test the relevance of these genes for 1,4-butanediol metabolism, several knockout strains were generated. The dehydrogenases encoded in the ped cluster (PP_2673-80) were constitutively expressed at a high level (Figure 3). To test the importance of these dehydrogenases to the degradation of 1,4-butanediol, the entire cluster (Δ*pedE-I*), as well as individual genes *pedE* and *pedI*, were knocked out in *P. putida* KT2440. When *P. putida* KT2440 Δ*pedE-I* was cultivated in MSM with 1,4-butanediol no growth could be observed, nor was the substrate taken up or converted to 4-hydroxybutyrate (Figure 4). Therefore, this cluster appears to be essential for the uptake and metabolism of 1,4-butanediol. The fact that no oxidation products were observed strongly suggests that these enzymes catalyze the initial oxidation steps.

The single knockouts of *pedE* and *pedI* were streaked on MSM plates containing 20 mM 1,4-butanediol as sole carbon source. Of these knockouts, *P. putida* KT2440 Δ*pedE* did not grow, while the Δ*pedI* strain displayed growth similar to the wildtype after 48 h (Figure 4). Thus, the PQQ-dependent alcohol dehydrogenase PedE is likely responsible for the oxidation of 1,4-butanediol, while, surprisingly, the aldehyde dehydrogenase PedI does not seem to play an essential role in the further oxidation steps, likely because of the high redundancy of aldehyde dehydrogenases in *P. putida*. PedE, a homolog to ExaA from *P. aeruginosa*, is an extensively investigated pyrroloquinoline quinone alcohol dehydrogenase with a broad substrate activity, including 1-butanol and 1,4-butanediol (Takeda et al., 2013). Furthermore, Wehrmann et al. (2017) showed activities of PedE toward structural similar alcohols and aldehydes of 1,4-butanediol, like 1-butanol and butyraldehyde. Additionally, the first steps of 1-butanol assimilation in *P. putida* BIRD-1 also involve homologs of the *ped* cluster (Simon et al., 2015; Vallon et al., 2015; Cuenca et al., 2016). The other dehydrogenases encoded within the *ped* cluster, PedH and also PedI, seem to be of minor relevance. PedE and PedH are both ethanol dehydrogenases but are inversely regulated by lanthanides. In the absent of those rare earth elements, *pedE* expression is induced and *pedH* is repressed (Wehrmann et al., 2017). Both PedE and PedH are highly expressed, but considering the absence of lanthanides, it is likely that PedH is not active.

The mutations found in PP_2046 and the upregulation of the adjacent operon PP_2047-51 strongly indicates an important role of the encoded enzymes. This operon contains an iron-containing alcohol dehydrogenase encoded by PP_2049 in addition to β-oxidation related genes. In literature, this dehydrogenase is placed in a context of β-oxidation, likely due to its association with the other genes in the PP_2047-51 operon (Poblete-Castro et al., 2012). However, PP_2049 is classified as an iron-containing alcohol dehydrogenase, which belongs to type III non-homologous NAD(P)^+^-dependent alcohol dehydrogenases (Mitchell et al., 2019). This family is known to have activity toward methanol, ethanol, propanol, and butanol (Hiu et al., 1987; Gaona-López et al., 2016). It is highly likely that PP_2049 oxidizes one or more of the alcohol groups of 1,4-butanediol. Thus, neither direct oxidation to succinate nor β-oxidation can be ruled out by the observed mutation in PP_2046 and the upregulation of the associated operon. In order to determine the relevance of the operon and to distinguish between the effect of the alcohol dehydrogenase and the β-oxidation related genes, the operon and individual genes PP_2046, PP_2049, and PP_2051 were knocked out in wildtype *P. putida* KT2440 and in the evolved strains B10.1 and B10.2. Care was taken to avoid polar effects in the in-operon knockouts by leaving start and stop codons of overlapping genes intact using the pEMG system (Martínez-García and de Lorenzo, 2011). Deletion strains were tested for their ability to grow on MSM with 1,4-butanediol or 4-hydroxybutyrate as sole carbon source (Figure 4).

**FIGURE 5:**
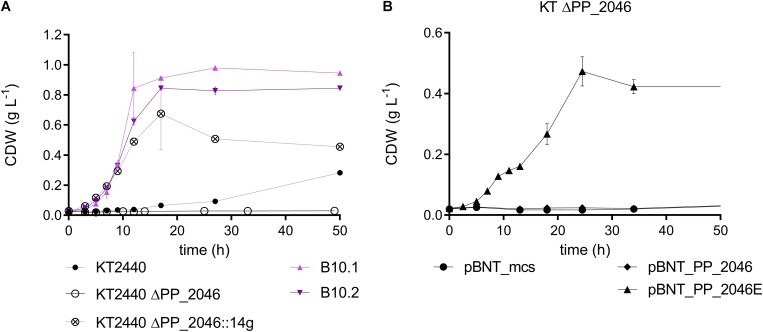
**(A)** Biomass growth during the cultivation of *P. putida* KT2440 (black, circles), B10.1, B10.2 (purple, triangles), *P. putida* KT2440 ΔPP_2046 (black, circle) and *P. putida* KT2440 ΔPP_2046:14g (black, circled cross) in MSM medium with 20 mM 1,4-butanediol. **(B)** Biomass growth of *P. putida* KT2440 ΔPP_2046 transformants harboring an overexpressing construct for PP_2046 or PP_2046E and the empty vector cultivated in MSM with 20 mM 1,4-butanediol. Error bars indicate the deviation of the mean (*n* = 2).

Both the wildtype and the evolved strains were unable to grow on 1,4-butanediol or 4-hydroxybutyrate when the regulator PP_2046, or the alcohol dehydrogenase PP_2049 were deleted (Figures 4, 5). The knockout of the whole operon also abolished growth. In contrast, deletion of PP_2051 did not affect growth on 1,4-butanediol (Supplementary Figure S3). This strongly suggests that PP_2049 is the main enzyme involved in the oxidation of 4-hydroxybutyrate. Both individual knockout strains of PP_2049 and *pedE* were unable to grow on 1,4-butanediol, making it unlikely that they oxidize the same substrate. More likely, the PP_2049 dehydrogenase is essential for the oxidation of 4-hydroxybutyrate, while PedE oxidizes 1,4-butanediol. Attempts to obtain direct biochemical evidence for the oxidation hypothesis with dehydrogenase assays on whole cell extracts of *P. putida* KT2440 wildtype, B10.1, and ΔPP_2046 with 4-hydroxybutyrate as a substrate were unsuccessful (Supplementary Figure S4) although this might be caused by instability of the PP_2049 enzyme and should be investigated further. The lack of phenotype of the PP_2051 mutant suggests that β-oxidation is not involved, however, this is no clear proof since several other acetyl-CoA C-acetyltransferases are also expressed on a similar level (Figure 3).

The deletion of PP_2046 apparently causes a downregulation of the operon, while the mutations in PP_2046 in the evolved strains cause an overexpression of the adjacent operon. This is evident in the proteome data and also described in Li et al. (2019) for the E34G mutation in the context of ethylene glycol metabolism. In fact, the strains evolved on ethylene glycol also grow efficiently on 1,4-butanediol (Supplementary Figure S5). Expression *in trans* of the mutated regulator from B10.2 containing this mutation (denoted as PP_2046E) in *P. putida* KT2440 ΔPP_2046 enhanced growth of the wildtype on 1,4-butanediol, while expression of the native version could not restore growth (Figure 5). The regulator PP_2046 groups in the LysR-type family, which mainly contains transcriptional activators, repressors, and even dual function activators/repressors with a helix-turn-helix (HTH) DNA-binding domain at the N-terminus (Pérez-Rueda and Collado-Vides, 2000; Maddocks and Oyston, 2008). Both mutations found in the B10.1 and B10.2 evolved strains are located in the first third of the gene, in the HTH domain. While the B10.1 version of PP_2046 lost its native start codon, alternatives start codons are present (Supplementary Figure S1). The fact that only the mutated version of PP_2046 can enable growth on 1,4-butanediol, while its deletion abolishes growth, strongly suggests that this gene encodes an activator of the downstream operon, with an unknown inducer outside of the 1,4-butanediol context. It seems that a modification of the HTH domain is key to creating a constitutive activator.

In order to test whether overexpression of the PP_2047-51 operon alone was sufficient to enable faster growth on 1,4-butanediol, PP_2046 was replaced by the strong constitutive promotor P_14__*g*_, facing the operon, resulting in the strain *P. putida* KT2440 ΔPP_2046:14g. Indeed, the growth on 1,4-butanediol was enhanced by 3.43-fold compared to the wildtype (Figure 5). This further indicates that the PP_2047-51 operon is the main determinant enabling fast growth on 1,4-butanediol. However, growth of this strain was somewhat slower than that of the evolved strains (ΔPP_2046:14g: 0.249 ± 0.004 h^–1^), indicating that other factors, possibly also regulated by PP_2046, are at play.

**FIGURE 6:**
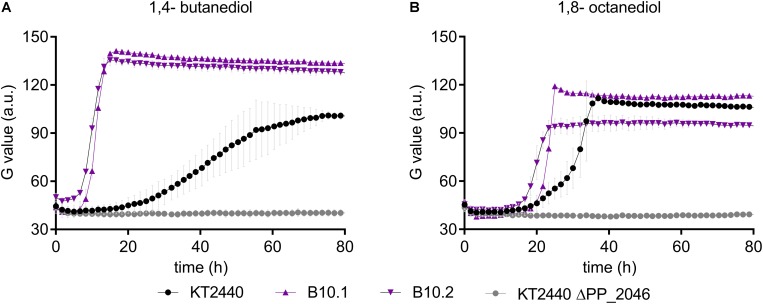
Growth of *P. putida* KT2440 (black, circles), B10.1, B10.2 (purple, triangles) and ΔPP_2046 (gray, circles) on 1,4-butanediol **(A,B)** 1,8-octanediol. Growth was detected via a Growth Profiler^®^ in 24-square well plates. Error bars depict the deviation from mean (*n* = 2).

In this context, it should be noted that no gene encoding a CoA-ligase or transferase is present within the operon, which would be required for 1,4-butanediol degradation through β-oxidation. However, several of such enzymes were upregulated in the presence of 1,4-butanediol (Figure 3). To test whether the upregulation of this operon in the evolved strains enables enhanced β-oxidation, growth of the wildtype, the evolved strains, and ΔPP_2046 were analyzed on longer-chain α,ω-diols. The ΔPP_2046 strain did not grow on any of the tested diols. In contrast, both the evolved strain and the wildtype grew on 1,4-butanediol and 1,8-octanediol, with the evolved strains growing at a significantly higher rate (Figure 6). Surprisingly, none of the strains grew on 1,6-hexanediol or 1,7-heptanediol. A similar trend of faster growth by the evolved strains was also observed on butanol (Supplementary Figure S6). Since these substrates can only be metabolized through β-oxidation, these results strongly suggest that the upregulation of the PP_2047-51 operon enables higher activity of this pathway, and they prove that PP_2046 is an essential regulator for the metabolism of these short- to medium-chain alcohols.

## Conclusion

Adaptive laboratory evolution was successfully used to enhance growth of *P. putida* KT2440 on 1,4-butanediol. Putative degradation pathways of this important plastic monomer were contextualized with leads from genome resequencing and proteome analysis, which were verified by knockout and overexpression analyses and physiological data. The alcohol dehydrogenases PedE and PP_2049 were found to be essential for growth on 1,4-butanediol, with the latter also being required for growth on 4-hydroxybutyrate. Mutations in the transcriptional regulator PP_2046 were the main cause of enhanced growth in the ALE strains. The evolved phenotype could be reproduced through reverse engineering, either by overexpression of the PP_2047-51 operon by promoter exchange, or through *in trans* expression of the mutated regulator. In all, the knockout analysis favors the hypothesis of direct oxidation of 1,4-butanediol, via 4-hydroxybutyrate, to succinate. However, the alternative β-oxidation hypothesis can’t be ruled out, and possibly both pathways operate simultaneously.

## Data Availability Statement

The sequences have been deposited in the NCBI Sequence Read Archive (SRA) with the accession number SRP148839 for ethylene glycol ALE strains (including our laboratory wildtype SRX4119395 used in this study) and SRP148839 for the 1,4-butanediol ALE strains. Raw data for all figures shown is available from the author upon reasonable request.

## Author Contributions

NW and LB conceived the study with the help of KO’C and TN. NW supervised the study with support of LB and KO’C. NW and W-JL designed the experiments. W-JL performed the experiments with the help of P-JN. TN performed the proteome analysis. W-JL and NW prepared the figures and wrote the manuscript with the help of all authors. All authors have read and approved the final version of this manuscript, analyzed, and interpreted the data. SK oversaw PHA production experiments.

## Conflict of Interest

SK was employed by the company Bioplastech Ltd. KO’C was employed by university college Dublin. He has a shareholding in the company Bioplastech Ltd. The remaining authors declare that the research was conducted in the absence of any commercial or financial relationships that could be construed as a potential conflict of interest.

## Footnotes

1 www.uniprot.org
