# Supplementary material for: Unraveling 1,4-Butanediol Metabolism in Pseudomonas putida KT2440
Source: Front Microbiol. 2020 Mar 17;11:382. doi: 10.3389/fmicb.2020.00382 (PMC7090098; doi:10.3389/fmicb.2020.00382)
Supplement: Supplementary file 1 [file Data_Sheet_1.PDF]

Supplemental data to:

Unraveling 1,4-butanediol metabolism in *Pseudomonas putida* KT2440

Wing-Jin Li[1], Tanja Narancic[2,3], Shane T. Kenny[4], Paul-Joachim Niehoff[1], Kevin O'Connor[2,3], Lars M. Blank[1], Nick Wierckx[1,5]*

[1] Institute of Applied Microbiology-iAMB, Aachen Biology and Biotechnology-ABBt, RWTH Aachen University, Aachen, Germany

[2] UCD Earth Institute and School of Biomolecular and Biomedical Science, University College Dublin, Belfield, Dublin 4, Ireland

[3] BEACON – SFI Bioeconomy Research Centre, University College Dublin, Belfield, Dublin 4, Ireland

[4] Bioplastech Ltd., NovaUCD, Belfield Innovation Park, University College Dublin, Belfield, Dublin 4, Ireland

[5] Institute of Bio- and Geosciences IBG-1: Biotechnology, Forschungszentrum Jülich, 52425 Jülich, Germany

*** Correspondence:** n.wierckx@fz-juelich.de

Table S1 Oligonucleotides used in this work

name	sequence	template	direc-tion	purpose
**MO48**	ACTATAGGGCGAATTGGAGC	*P. putida* KT2440	rw	pBNT
**WJ49**	GCTCGGTACCCGGGGATCCTCTAGAGAATTCAGTACTGGTGGCCGAAGA	*P. putida* KT2440	fw	pEMG_ΔPP_0411
**WJ50**	GCAAGGATCCCCTAGGGGGGGTACTGAGAGAATG	*P. putida* KT2440	rw	pEMG_ΔPP_0411
**WJ51**	CAGTACCCCCCCTAGGGGATCCTTGCCTGTACCGGCCTCTTC	*P. putida* KT2440	fw	pEMG_ΔPP_0411
**WJ52**	TGCATGCCTGCAGGTCGACTCTAGAGTCGACCAGCGTCCCCGGGAACAG	*P. putida* KT2440	rw	pEMG_ΔPP_0411
**WJ54**	AGATTGAGCTGGTACGTGAG	*P. putida* KT2440	fw	pEMG_ΔPP_0411
**WJ55**	GCATAAGCGTCCATGAACAG	*P. putida* KT2440	rw	pEMG_ΔPP_0411
**M13uni (-43)**	AGGGTTTTCCCAGTCACGACGTT	pEMG	fw	pEMG
**M13rev (-49)**	GAGCGGATAACAATTTCACACAGG	pEMG	rw	pEMG
**WJ56**	TCTCGGTACCCGCTGGCCCTTAACATTCCC	*P. putida* KT2440	fw	pEMG_Δ*pedE*
**WJ57**	CCGCTCTAGAAATTTCCACCCGCTATTCAC	*P. putida* KT2440	rw	pEMG_Δ*pedE*
**WJ58**	TACATCTAGACACCTCAATTGGCCCTTCGC	*P. putida* KT2440	fw	pEMG_Δ*pedE*
**WJ59**	ATTCGTCGACTTGTACACCGCCACCTTGAG	*P. putida* KT2440	rw	pEMG_Δ*pedE*
**WJ60**	TCTCGAGCTCCTCACCGACAAGCCGGTAG	*P. putida* KT2440	fw	pEMG_Δ*pedH*
**WJ61**	TCTCGGATCCTCTTGGTCCCGACCCGATTG	*P. putida* KT2440	rw	pEMG_Δ*pedH*
**WJ62**	TCTCGGATCCCGGCCCTACTACCAAATGAC	*P. putida* KT2440	fw	pEMG_Δ*pedH*
**WJ63**	TCTCGTCGACTCTTGGCAATGCGCTTGCTG	*P. putida* KT2440	rw	pEMG_Δ*pedH*
**WJ64**	TCTCGGTACCACGTGCTCGACCGCACCAAC	*P. putida* KT2440	fw	pEMG_Δ*pedI*
**WJ65**	ATCGTCTAGATTTGGTAGTAGGGCCGCTTG	*P. putida* KT2440	rw	pEMG_Δ*pedI*
**WJ66**	ATCGTCTAGAGCCCGCTCCCACAGGTTCAC	*P. putida* KT2440	fw	pEMG_Δ*pedI*
**WJ67**	ATCGGTCGACGGCACCAAAGATGATTTCAG	*P. putida* KT2440	rw	pEMG_Δ*pedI*
**WJ68**	GGGCTTGCGCCTGTTCATTC	*P. putida* KT2440	fw	sequencing *pedE*
**WJ69**	GCTGTGTACAGGCAGTAGTC	*P. putida* KT2440	rw	sequencing *pedI*
**WJ74**	TCTCGAATTCTCCGGCATCCACCTGGCCTC	*P. putida* KT2440	fw	pEMG_ΔPP_2046
**WJ75**	TCTCGGTACCAGCCATCAGGAAACGCGATAG	*P. putida* KT2440	rw	pEMG_ΔPP_2046

name	sequence	template	direc-tion	purpose
**WJ76**	TCTCGGTACCTACCTTCGGCCTGCTTAGGG	*P. putida* KT2440	fw	pEMG_ΔPP_2046
**WJ77**	TTCCTCTAGATCCAGGTCGATGCCCACCAC	*P. putida* KT2440	rw	pEMG_ΔPP_2046
**WJ80**	TGAGGCTGACAGTGGCATTG	*P. putida* KT2440	fw	sequencing PP_2046
**WJ81**	AGCGCATTATCGACCTGCAC	*P. putida* KT2440	rw	sequencing PP_2046
**WJ93**	AGGTACCGAATTCCTCGAGTTAGGAGGTATTTCGTATGCCATATATTTTCAGCATGAATATTTCGAACTTCGACCTGAACCTG	*P. putida* KT2440 E12 evo1	fw	pBNT_PP_2046E
**WJ94**	GCCCGACGTCGCATGCTCCTTCTAGATCAGGCGGGGGGGAGCGT	*P. putida* KT2440 E12 evo1	rw	pBNT_PP_2046E
**WJ95**	ACGCTCCTGCTTTCTTGTAG	*P. putida* KT2440	rw	sequencing PP_2046
**WJ96**	GAATAGCGGGTGGAAATTGG	*P. putida* KT2440	fw	sequencing *pedE*
**WJ102**	TTCCGAATTCTGCATGCCCTGGCCTATCCG	*P. putida* KT2440	fw	pEMG_ΔPP_2051
**WJ103**	TTCCAGGTACCCTTTCATGATGGCTGTTCC	*P. putida* KT2440	rw	pEMG_ΔPP_2051
**WJ104**	TTCCGGTACCCAAGGCCAGCCCATGGCGCTGAC	*P. putida* KT2440	fw	pEMG_ΔPP_2051
**WJ105**	TTCCTCTAGACAGCAGCGCCATGAGCCAGC	*P. putida* KT2440	rw	pEMG_ΔPP_2051
**WJ106**	GCTGCTGGCGGATAACCTTG	*P. putida* KT2440	fw	mapping PP_2051
**WJ107**	GCACACGCAAATCTTCAACG	*P. putida* KT2440	rw	mapping PP_2051
**WJ119**	AGCTCGGTACCCGGGGATCCTCCGGCATCCACCTGGCCTC	*P. putida* KT2440	fw	pEMG_ΔPP_2046::14g
**WJ120**	CCTAGGTCGTGCAATTATACCTGGCCGCGAGAGCCTTGTCAATGGGCTTAATTAAAGCCATCAGGAAACGCGATAG	*P. putida* KT2440	rw	pEMG_ΔPP_2046::14g
**WJ121**	TTAATTAAGCCCATTGACAAGGCTCTCGCGGCCAGGTATAATTGCACGACCTAGGTACCTTCGGCCTGCTTAGGG	*P. putida* KT2440	fw	pEMG_ΔPP_2046::14g
**WJ122**	TGCATGCCTGCAGGTCGACTTCCAGGTCGATGCCCACCAC	*P. putida* KT2440	rw	pEMG_ΔPP_2046::14g
**WJ129**	GCTCGGTACCCGGGGATCCTGATCCGATCATCGTCCATC	*P. putida* KT2440	fw	pEMG_ΔPP_2049
**WJ130**	CCTCATAGATCGCACTCTCCTTGTTCGTG	*P. putida* KT2440	rw	pEMG_ΔPP_2049
**WJ131**	GGAGAGTGCGATCTATGAGGCAGCCTACTGATG	*P. putida* KT2440	fw	pEMG_ΔPP_2049
**WJ132**	TGCATGCCTGCAGGTCGACTTCCTGACCTGCGCCAATG	*P. putida* KT2440	rw	pEMG_ΔPP_2049
**WJ134**	GATCGAATTCCCAGATGTGCCGCAAGCCCAG	*P. putida* KT2440	fw	pEMG_ΔPP_2047
**WJ135**	GTATGGTACCTCCAGCCACAGCACCGACAG	*P. putida* KT2440	rw	pEMG_ΔPP_2047
**BW13**	TTTGCACTGCCGGTAGAAC	pEMG	fw	pEMG MCS mapping

name	sequence	template	direc-tion	purpose
**BW14**	AATACGCAAACCGCCTCTC	pEMG	rw	pEMG MCS mapping

Table S2 Plasmids used in this work

plasmid	genotype	reference
pRK2013	Km^r, oriV(RK2/ColE1), mob^+, tra^+	Figurski and Helinski, 1979
pSW-2	Gm^r, oriRK2, xylS, Pm→I-sceI (transcriptional fusion of I-*sceI* to Pm)	Martínez-García and Lorenzo, 2011
**pEMG and derivatives**		
pEMG	Kan^r, oriR6K, lacZ α wth two flanking I-SceI sites	Martínez-García and Lorenzo, 2011
pEMG_Δ*gcl*	pEMG bearing flanking sequences of *gcl*, *gcl* deletion delivery vector	this work
pEMG_Δ*gclR*	pEMG bearing flanking sequences of *gclR*, *gclR* deletion delivery vector	this work
pEMG_Δ*pedE*	pEMG bearing flanking sequences of *pedE*, *pedE* deletion delivery vector	Li et al., 2019
pEMG_Δ*pedE-I*	pEMG bearing flanking sequences of *pedE-I*, *pedE-I* deletion delivery vector	Li et al., 2019
pEMG_Δ*pedH*	pEMG bearing flanking sequences of *pedH*, *pedH* deletion delivery vector	Li et al., 2019
pEMG_Δ*pedI*	pEMG bearing flanking sequences of *pedI*, *pedI* deletion delivery vector	Li et al., 2019
pEMG_ΔPP_0411-0413	pEMG bearing flanking sequences of PP_0411-13, PP_0411-13 deletion delivery vector	this work
pEMG_ΔPP_2046	pEMG bearing flanking sequences of PP_2046, PP_2046 deletion delivery vector	this work
pEMG_ΔPP_2046::14g	pEMG bearing flanking sequences of PP_2046 and integration of the synthetic promotor 14g, replacing PP_2046 with 14g delivery vector	this work
pEMG_ΔPP_2047-51	pEMG bearing flanking sequences of PP_2047-51, PP_2047-51 deletion delivery vector	this work
pEMG_ΔPP_2049	pEMG bearing flanking sequences of PP_2049, PP_2049 deletion delivery vector	Niehoff (2017)
pEMG_ΔPP_2051	pEMG bearing flanking sequences of PP_2051, PP_2051 deletion delivery vector	this work

**plasmid**	**genotype**	**reference**
pEMG_ΔPP_2662	pEMG bearing flanking sequences of PP_2662, PP_2662 deletion delivery vector	this work
pEMG_ΔPP_2662::14d	pEMG bearing flanking sequences of PP_2662 and integration of the synthetic promotor 14d, replacing PP_2662 with 14d delivery vector	this work
**Expression vectors**		
**pBNT and derivatives**		
pBNT	Km^r, P_{nagAa}: nag promoter without RBS, salicylate-inducible	Verhoef et al., 2010
pBNT_PP_2046	pBNT_(MCS) plasmid with PP_2046 from *P. putida* KT2440	this work
pBNT_PP_2046E	pBNT_(MCS) plasmid with evolved PP_2046 from *P. putida* KT2440, E6.1	this work

Table S3 List of mutations (Single Nucleotide Polymorphisms (SNP) and Insertion-Deletion polymorphisms (InDel)) found in the genome of the evolved strains B10.1 (not underlined) or B10.2 (underlined) but not in our laboratory *P. putida* KT2440. The mutated gene found in both evolved strains, but not in the wildtype, is highlighted in bold.

**type**	**strain**	**position**	**locus tag**	**codon change**	**functional class**	**annotation**
SNP	B10.2	196495	PP_0168	acG/acC	silent	surface adhesion protein
	B10.1	197524	PP_0168	gtA/gtG	silent	
	B10.1	197551	PP_0168	acC/acG	silent	
	B10.2	197551	PP_0168	acC/acG	silent	
	B10.1	197572	PP_0168	aaG/aaA	silent	
	B10.2	197572	PP_0168	aaG/aaA	silent	
	B10.1	197590	PP_0168	gaT/gaC	silent	
	B10.2	197590	PP_0168	gaT/gaC	silent	
	B10.2	698939	PP_16SD	NA	none	rRNA
	**B10.2**	**2328228**	**PP_2046**	**gAg/gGg**	**missense**	**lysR family regulator**
	**B10.1**	**2328326**	**PP_2046**	**atG/atA**	**nonsense**	**lysR family regulator**
	B10.2	3287225	PP_2889	gCg/gGg	missense	transmembrane anti-sigma factor
	B10.1	4345003	PP_3818	gaA/gaG	silent	OmpA/ MotB domain-containing protein
	B10.2	4348955	intergenic	NA	none	

type	strain	position	locus tag	codon change	functional class	annotation
**InDel**	B10.1	2443249	PP_2139	GTGCGCC GCTGGTG CTGGAGA TTGTGCC GCACAAG CATGAGA TCGACCC GAAGTAC CACTTCC TGTGCGA --> G	none	DNA topoisomerase I - topA

ATA→Start loss

TTTTTGAAATTTCCATTTCGTATGCCATATATTTTCAGCATGAATATTTCGAACTTCGACCT
GAACCT   GGG→E34G
GCTGCGCGTCTTCGACATGTTGCTGCGTGAACAGAATGTATCCCGGCAGCCGCGCGTCTGG
CCCTGACCCAGCCGACCGTGAGCAATGCCCTGGCGCGCCTGCGTGACCAGCTGGGTGACCCG
CTGCTGGTCCGCGTGGGCCGGCGCATGCGCCCGACGCCACGGGCCTTGGCACTGGAGGGGCC
GATACGTGCGGCGTTACAGCAGATCGAGCAGACGCTGGGCACCGGCGATGGTTTCGAGCCTC
AGCGCAGCCATCGCCAGCTGCGCATCGCCCTCACCGATTTCGTCGAACAGCTGTGCATGCCG
CCACTCCTGGCGCGGCTGGAGCTACTGGCACCCAACGTGCGCATCGACGTGGTGCACCTGGC
CCCCAACCTGCCGGCCGAGGCGCTGGACCGGGGCGACCTCGACCTGGTACTGGGCCGTTTCG
ACGAGGTGCCGGCGCGCTTCACCCGCCACCCCTGGCGCCGTGAAACCCTGCAGATCGCGCTG
CGCCAGCAGCACCCGCACCTGGCGCCGGGCCAGGCACTGGACCTCGACGCATTCCTGGGCTT
GCGGCACATCTGGGTGCACGGCGGCCAGACCCGGGGCATGGTCGACCAGTGGCTGGCCGAGC
AAGGCCTGACCCGGCAAATCGCCTATACCACGCCCAACTACCTGCAGGCCGCCCATCTGGCC
GCAGCCACCGACATGTGTGTGGTGCTGCCGCGGCAACTGGCGCAGCAGTTTGCGCACCTGCT
GCCATTGGCGGTGCACGAACTGCCATTTGCCCTGGAGCCTTTCGAATTGGAAGTGGTGCACC
TGAGCCACCGTCAGCACGACCCCGCCCTGGCCTGGCTGGTCGAACAGATCCTCACGCTCCCC
CCCGCCTGAAGCCATCAGGAAACGCGATA

Figure S1 Sequence of PP_2046 from *P. putida* KT2440. Native (red) and alternative in-frame (orange) start codons, as well as the mutations in B10.1 (ATG → ATA: start loss) and B10.2 (GCG – GGG: E34G) (blue) are shown. The underlined sequence encodes a putative helix-turn-helix DNA binding domain (Letunic and Bork, 2018).

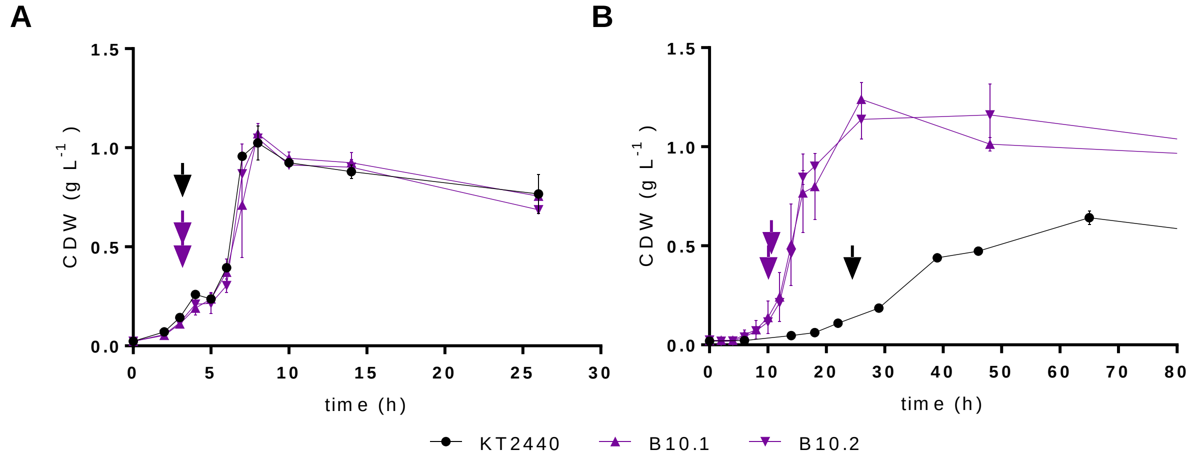

Figure S2 Biomass growth of *P. putida* KT2440 and the evolved strains B10.1 and B10.2 cultivated in MSM with 13.3 mM glucose (**A**) or 20 mM 1,4-butanediol (**B**). Arrows indicate the time when samples were taken for proteome analysis. Error bars indicate the standard deviation (n = 3).

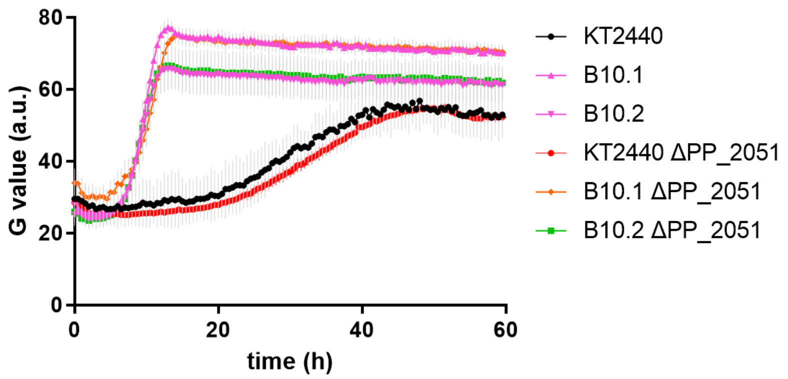

Figure S3 Growth of *P. putida* KT2440 (black, circles), B10.1, B10.2 (purple, triangles) and the respective ΔPP_2051 knockouts in KT2440 (red, circles), B10.1 (orange, diamonds) and B10.2 (green, squares) in MSM with 20 mM 1,4-butanediol. Growth was detected via a Growth Profiler® in 24-square well plates. Error bars depict the standard error of the mean (n = 3).

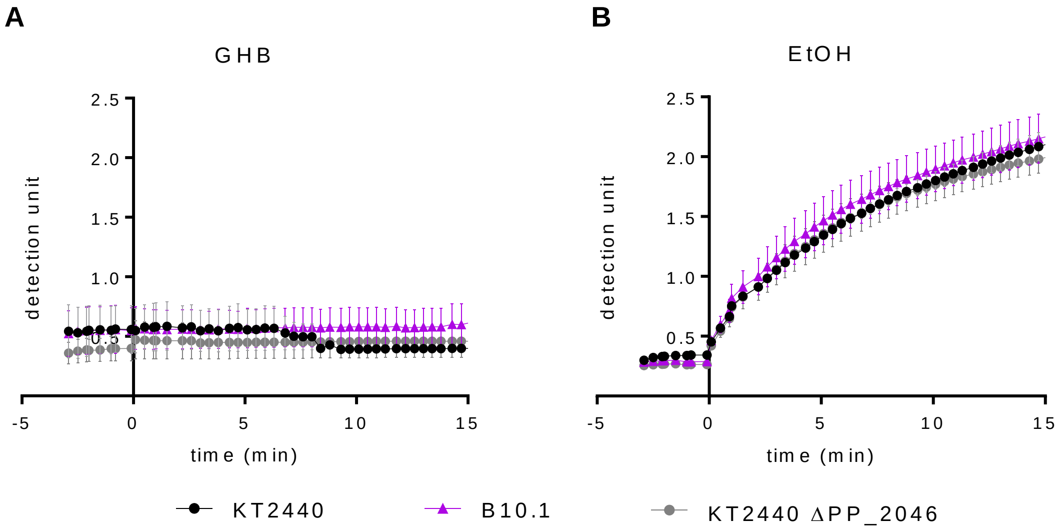

Figure S4 Dehydrogenase activity assay with 4-hydroxybutyrate (GHB) (**A**) or ethanol (EtOH) (**B**) as substrate. Crude cell extracts obtained from *P. putida* KT2440 (black, circles), B10.1, B10.2 (purple, triangles) and *P. putida* KT2440 ΔPP_2046 (grey, circles) Error bars depict the standard error of the mean (n = 2-3).

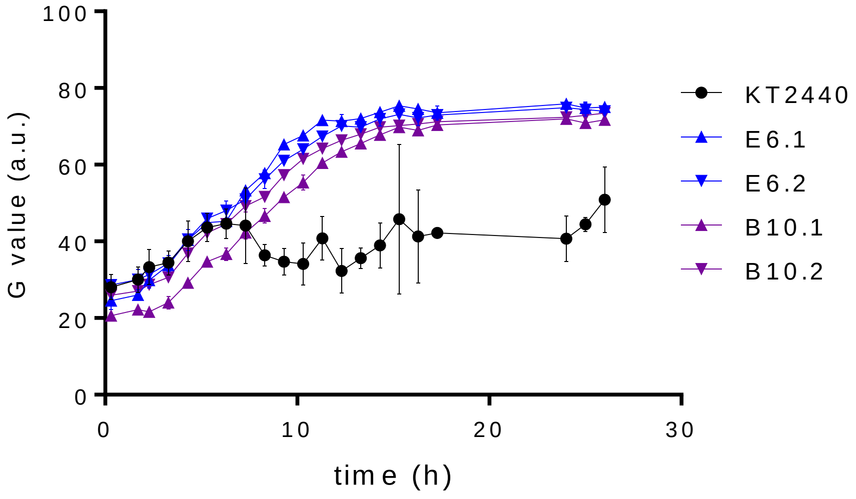

Figure S5 Growth comparison of wildtype *P. putida* KT2440, strains E6.1 and E6.2 evolved on ethylene glycol (Li et al., 2019), and the strains B10.1 and B10.2 evolved on 1,4-butanediol, cultivated in MSM with 20 mM 1,4-butanediol. Growth was detected via the Growth Profiler® in a 24-well plate. Error bars indicate the standard deviation (n=3).

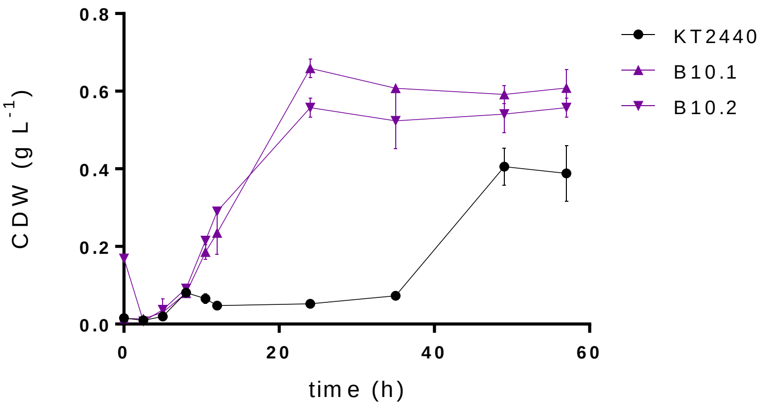

Figure S6 Biomass growth of *P. putida* KT2440 (black, circles) and the evolved strains B10.1 (purple, triangle) and B10.2 (purple, inverted triangle) in shake flasks in MSM with 20 mM 1-butanol. Error bars indicate the deviation of the mean (n = 2).

REFERENCES

Figurski, D. H., and Helinski, D. R. (1979). Replication of an origin-containing derivative of plasmid RK2 dependent on a plasmid function provided in trans. *Pro Natl Acad Sci USA* 76, 1648–1652. doi: 10.1073/pnas.76.4.1648

Letunic, I., and Bork, P. (2018). 20 years of the SMART protein domain annotation resource. *Nucleic acids research* 46, D493-D496. doi: 10.1093/nar/gkx922

Li, W.-J., Jayakody, L. N., Franden, M. A., Wehrmann, M., Daun, T., Hauer, B., et al. (2019). Laboratory evolution reveals the metabolic and regulatory basis of ethylene glycol metabolism by *Pseudomonas putida* KT2440. *Environ Microbiol*. doi: 10.1111/1462-2920.14703

Martínez-García, E., and Lorenzo, V. de (2011). Engineering multiple genomic deletions in Gram-negative bacteria: Analysis of the multi-resistant antibiotic profile of *Pseudomonas putida* KT2440. *Environ Microbiol* 13, 2702–2716. doi: 10.1111/j.1462-2920.2011.02538.x

Verhoef, S., Ballerstedt, H., Volkers, R. J. M., Winde, J. H. de, and Ruijssenaars, H. J. (2010). Comparative transcriptomics and proteomics of p-hydroxybenzoate producing *Pseudomonas putida* S12: novel responses and implications for strain improvement. *Appl Microbiol Biotechnol* 87, 679–690. doi: 10.1007/s00253-010-2626-z
